# Distribution of Stromal Cell Subsets in Cultures from Distinct Ocular Surface Compartments

**DOI:** 10.18502/jovr.v15i4.7780

**Published:** 2020-10-25

**Authors:** Lei Liu, Ying Yu, Qiuyue Peng, Simone R Porsborg, Frederik M Nielsen, Annemette Jørgensen, Anni Grove, Chris Bath, Jesper Hjortdal, Ole B Christiansen, Trine Fink, Vladimir Zachar

**Affiliations:** ^1^Regenerative Medicine Group, Department of Health Science and Technology, Aalborg University, Aalborg, Denmark; ^2^Department of Neurosurgery, First Hospital of Jilin University, Changchun, China; ^3^Department of Gynecology and Obstetrics, Aalborg University Hospital, Denmark; ^4^Department of Pathology, Aalborg University Hospital, Denmark; ^5^Department of Ophthalmology, Aalborg University Hospital, Aalborg, Denmark; ^6^Department of Ophthalmology, Aarhus University Hospital, Aarhus, Denmark

**Keywords:** CD146, CD34, Flow Cytometry, Human Ocular Surface Stromal Cells, Pericytes

## Abstract

**Purpose:**

To reveal the phenotypic differences between human ocular surface stromal cells (hOSSCs) cultured from the corneal, limbal, and scleral compartments.

**Methods:**

A comparative analysis of cultured hOSSCs derived from four unrelated donors was conducted by multichromatic flow cytometry for six distinct CD antigens, including the CD73, CD90, CD105, CD166, CD146, and CD34.

**Results:**

The hOSSCs, as well as the reference cells, displayed phenotypical profiles that were similar in high expression of the hallmark mesenchymal stem cell markers CD73, CD90, and CD105, and also the cancer stem cell marker CD166. Notably, there was considerable variation regarding the expression of CD34, where the highest levels were found in the corneal and scleral compartments. The multi-differentiation potential marker CD146 was also expressed highly variably, ranging from 9% to 89%, but the limbal stromal and endometrial mesenchymal stem cells significantly surpassed their counterparts within the ocular and reference groups, respectively. The use of six markers enabled investigation of 64 possible variants, however, just four variants accounted for almost 90% of all hOSSCs, with the co-expression of CD73, CD90, CD105, and CD166 and a combination of CD146 and CD34. The limbal compartment appeared unique in that it displayed greatest immunophenotype diversity and harbored the highest proportion of the CD146+CD34- pericyte-like forms, but, interestingly, the pericyte-like cells were also found in the avascular cornea.

**Conclusion:**

Our findings confirm that the hOSSCs exhibit an immunophenotype consistent with that of MSCs, further highlight the phenotypical heterogeneity in stroma from distinct ocular surface compartments, and finally underscore the uniqueness of the limbal region.

##  INTRODUCTION

Continuous maintenance of the cornea is imperative to preserve the normal vision. It has been shown that the transitional zone between cornea and sclera known as limbus plays an important role,^[1,2]^ and we have in our previous work highlighted some important aspects of the epithelial stem cells associated with this part of the ocular surface.^[3,4,5]^ In addition to the limbal epithelial stem cells (LESCs), the limbus harbors a group of stem cell-like stromal cells capable of multilineage differentiation.^[6,7]^ Furthermore, it has been found that several subtypes of human ocular surface stromal cells (hOSSCs) display stem cell-like properties, with similar stem cell-like features being attributed to stromal cells from avascular central cornea^[8]^ and the sclera.^[9]^ Upon injury, the hOSSCs become mitotic, exhibit limited capacity for self-renewal and transit into myofibroblast phenotype.^[10,11]^ It has also been revealed that hOSSCs have a phenotype in common with mesenchymal stem cells (MSCs), including the expression of CD29, CD54, CD71, CD90, CD105, CD106, and CD166.^[8,9,12]^ The hOSSCs thus appear to represent a highly functional population, which likely plays an important role in the maintenance of cornea, and in the future may support a new generation of improved therapies for sight-threatening corneal diseases, such as limbal stem cell deficiency or corneal scarring.

Nevertheless, among the different ocular surface compartments, the co-expression of individual MSC markers, and, importantly, the specific phenotypical variants, such as pericyte- or adventitia-like cells, remains obscure. To this end, we embarked to isolate and culture the corneal (CSCs), limbal (LSCs), and scleral (SSCs) stromal cells from each of the four individuals and carry out six-epitopes multicolor immunophenotyping using flow cytometry with reference to the well-established adipose-derived stem cells (ASCs), endometrial mesenchymal stem cells (EMSCs), and human foreskin fibroblasts (HFFs). We were especially meticulous to use consistent dissection and culture techniques so as to eliminate possible confounding factors from inter-donor variability^[13]^ and culture conditions.^[14,15,16]^ The results are expected to deepen our understanding of the hOSSCs phenotypical diversity and spur further research into biological significance of specific variants for corneal homeostasis.

##  METHODS

### Ocular surface tissue dissection and cell culture

The tissue dissection and culture techniques were optimized based on our previous studies.^[4]^ Human ocular surface tissue without a corneal pathology but not suitable for transplantation was obtained from four donors free from ocular disease aged 22–86 years from the Department of Ophthalmology, Aarhus University Hospital (Arhus, Denmark) adhering to the Danish legislation pertinent to tissue donation. After the removal of epithelial and endothelial cell layers by mechanical scraping, the ocular surface tissue was further separated into corneal, limbal, and scleral compartments under a stereo dissection microscope (Nikon SMZ-2B; Nikon, Tokyo, Japan). The compartments were next morcellated and placed in a mixture of collagenase type IV and dispase II (both from Life Technologies, Naerum, Denmark) at 100 IU/mL and 2.4 IU/mL, respectively, in PBS at 37°C for 2 hours. The digested explants were cultured in 6-well culture plates in DMEM supplied with 10% FCS and 1% PNC/streptomycin (all from Life Technologies). Once the outgrowing cells reached the well edge, they were passaged from each well into a T75 flask (P1) together with the tissue pieces, which would not attach and were discarded during the next media removal. The culture medium was changed every two–three days and the cells were passaged at 80% confluence at a 1:3 ratio until passage 3.

ASCs were obtained from in-house frozen stocks (P2–P4) of Regenerative Medicine Group. Aalborg University (Aalborg, Denmark) and cultured according to previous reports.^[17,18]^ HFFs were cultured as previously described.^[19]^ EMSCs were isolated from human endometrium tissue after hysterectomy was kindly donated by the Department of Gynecology and Obstetrics, Aalborg University Hospital according to the Danish legislature, following previously reported protocol.^[20]^

### Flow Cytometry

Co-expression of six epitopes, including the CD34, CD73, CD90, CD105, CD146, and CD166 was analyzed on trypsinized hOSSCs and reference cells using a batch of directly conjugated antibodies (all from BD Bioscience, Albertslund, Denmark) using the CytoFLEX and the Kaluza 1.3 software package (both from Beckman Coulter, Copenhagen, Denmark) as previously described.^[21,22]^ Compensation values were established for each run to control for the bleed-through utilizing the BD CompBeads Set (BD Bioscience) and the AutoComp Wizard in Summit 6.1 (Beckman Coulter), and the Kaluza 1.3 when analyzing the data. The gating protocol included a demarcation of each cell population from the cellular debris followed by the determination of the area of stable flow and selection of single cells. A cut-off value representing the top 2.5 percentile of the fluorescence minus one (FMO) control was then used to establish the positive population for each of the markers.^[23,24]^

### Statistical analysis 

The data are presented as a Mean ± standard deviation (SD) derived from three independent experiments entailing three to four biological replicates of hOSSCs and three technical replicates of reference cell lines. Statistical analysis was performed within hOSSCs and within the reference cells, respectively. Routines from the SPSS 24.0 package (SPSS, Chicago, IL) included Bartlett's test for equality of variances, one-way analysis of variance (ANOVA) together with post-hoc Tukey's Honestly Significant Difference (HSD) test or non-parametric Mann–Whitney U test or Kruskal–Wallis test in case of multi-sample comparisons. *P* < 0.05 was considered as statistically significant.

##  RESULTS 

### Cell Morphology and Single Surface Markers Expression

**Figure 1:**
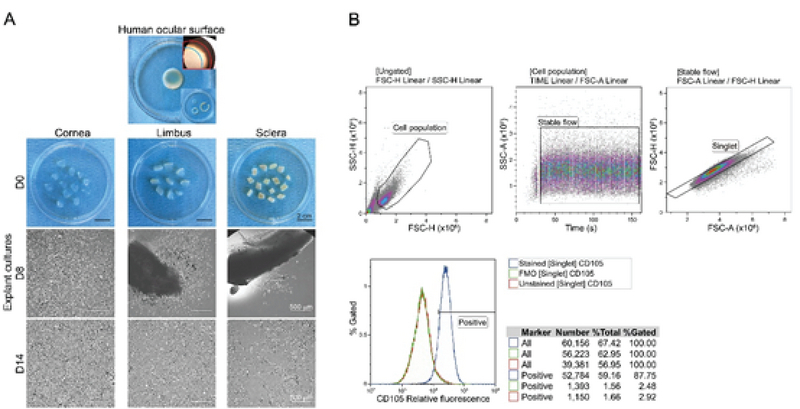
Isolation of human ocular surface stromal cells and outline of gating strategy to determine single maker positive boundary. **(A)** Donor corneal button with indicated (upper corner inset) and dissected (lower corner inset) corneal, limbal, and scleral compartments. The blue line demarcates cornea and limbus and the red line limbus and sclera (×20 original magnification). The representative images from explant cultures at days 0, 8, and 14 are shown. **(B)** Steps to identify positive populations in the flow cytometric analysis are demonstrated in the example of the CD105.

A representative corneal button as well as the isolated corneal, limbal, and scleral compartments and the explants thereof are shown in Figure 1A. Cell adhesion to the culture plates as outgrowth from the grafts was detected after 7–10 days. The outgrowing cells were morphologically typical of fibroblasts, assuming an elongated or spindle shape with a single nucleus and formed a monolayer by two weeks. The strategy to identify positivity boundaries for the studied markers in the flow cytometry is exemplified using the CD105 in Figure 1B. It demonstrates the application of gates to remove the cellular debris, select the stable flow and single cells, and determine the cut-off value using the FMO control distribution.

**Figure 2:**
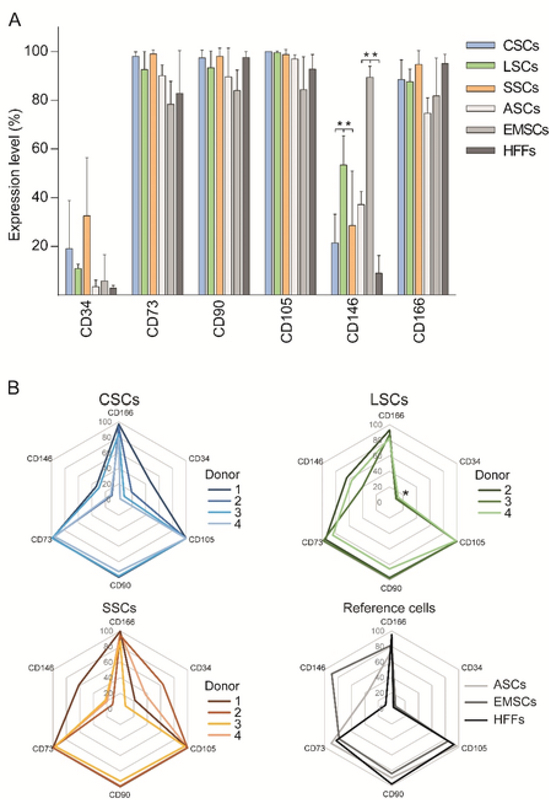
Immunophenotypical profiles of human ocular surface stromal cells with reference to mesenchymal stem cells. **(A)** Expression levels of single markers. **(B)** Radar charts indicating inter-donor variability. The data are presented as Means ± SD. The asterisks in panel A indicate a statistically significant (*p* < 0.05) difference between the means, and the asterisk in panel B denotes a significantly lower variability (*p* < 0.05) of the CD34 in the LSC group as compared to the CSCs and SSCs. *N* = 4 for CSCs and SSCs, and *n* = 3 for LSCs, ASCs, EMSCs, and HFFs. CSCs, corneal stromal cells; LSCs, limbal stromal cells; SSCs, scleral stromal cells; ASCs, adipose-derived stem cells; EMSCs, endometrial mesenchymal stem cells; HFFs, human foreskin fibroblasts.

**Figure 3:**
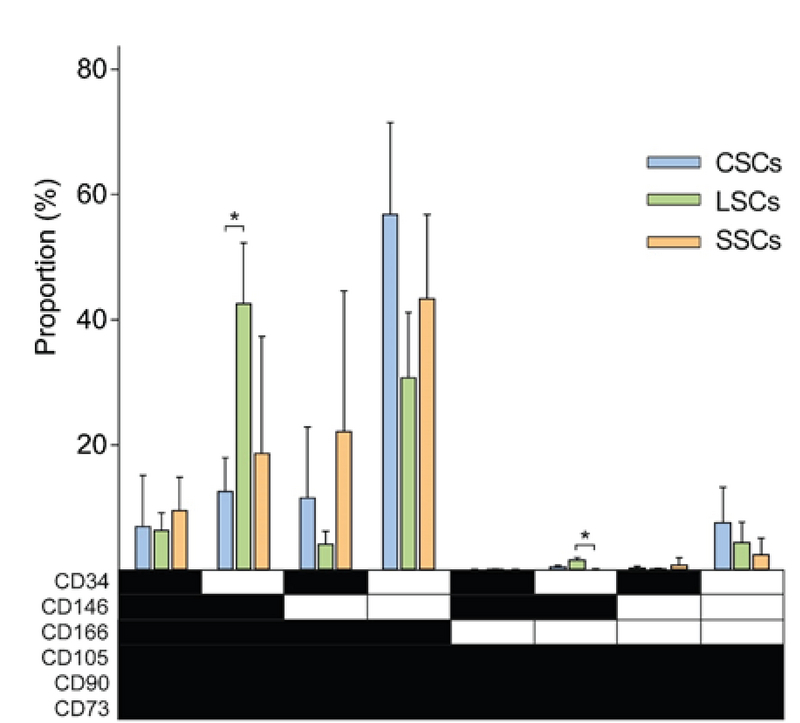
Distribution of phenotypical variants based on co-expression of six selected CD markers in stromal cells cultured from three distinct ocular surface compartments. The data are presented as Means ± SD and asterisks indicate statistical significance at *p* < 0.05. *N* = 4 for CSCs and SSCs, and *n* = 3 for LSCs. ▪ The marker is expressed. □ The marker is not expressed. CSCs, corneal stromal cells; LSCs, limbal stromal cells; SSCs, scleral stromal cells.

**Figure 4:**
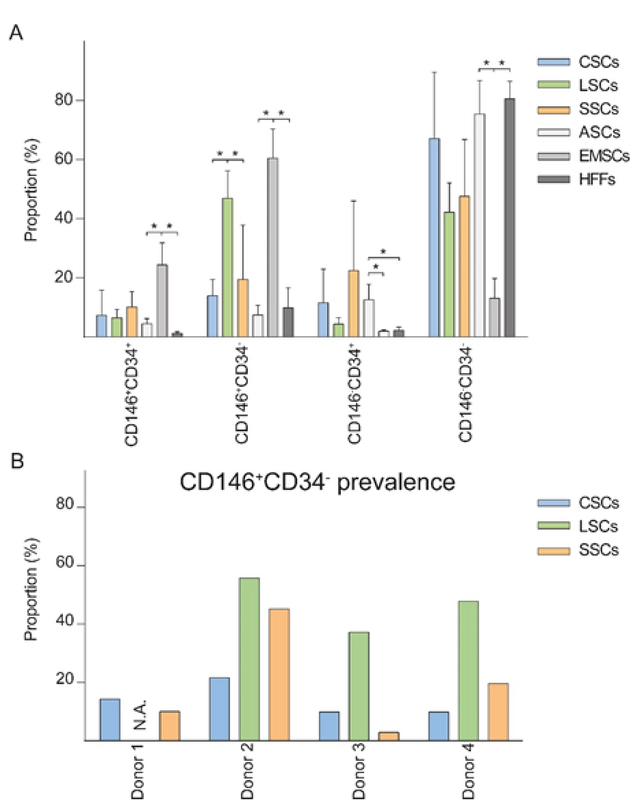
Co-expression of the CD146 and CD34 in human ocular surface stromal cells with reference to mesenchymal stem cells and fibroblasts. **(A)** Occurrence of pericytic (CD146+CD34–), adventitial (CD146–CD34+), and intermediate-like (CD146+CD34+) phenotypical variants. The data are presented as Means + SD and asterisks indicate statistical significance at *p* < 0.05. *N* = 4 for CSCs and SSCs, and *n* = 3 for LSCs, ASCs, EMSCs, and HFFs. **(B)** Inter-donor variability of the CD146+CD34– pericyte-like phenotype. CSCs, corneal stromal cells; LSCs, limbal stromal cells; SSCs, scleral stromal cells; ASCs, adipose-derived stem cells; EMSCs, endometrial mesenchymal stem cells; HFFs, human foreskin fibroblasts; N.A., not available.

The expression of hOSSC surface markers with reference to the ASCs, EMSCs, and HFFs is presented in Figure 2A. In general, all cell types expressed highly and uniformly the CD73, CD90, CD105, and CD166, with means ranging from 74.6% to 100%, but the expression of CD34 and CD146 was relatively lower and more variable. The means ranged from 2.7% to 32.5% for the CD34 and from 8.9% to 89.4% for the CD146. Only the CD146 was expressed significantly higher by the LSCs and EMSCs within the hOSSC and reference cell groups, respectively, and it is notable that it was the EMSCs that expressed this marker at the highest level (*p* < 0.01) when taking all the cell types into account.

A donor-matched phenotypic analysis of the ocular compartments revealed marked inter-donor variability of the CD146 expression (Figure 2B). Across the donors, the expression of CD146 varied from 10.4% to 33.5% in CSCs, 40.2% to 63.7% in LSCs, and 11.3% to 61.4% in SSCs. Interestingly, with respect to the CD34, the limbal compartment exhibited significantly the least variability with 9.6% to 12.9% of positive cells, whereas, the remaining two segments were more reminiscent of the CD146 pattern. The values namely ranged from 1.9% to 46.8% in CSCs and 7.6% to 63.4% in SSCs.

### Distribution of Phenotypical Variants

The combination of the studied six surface markers provided for 64 (2${}^{\wedge }$6) possible immunophenotypical variants. However, only a restricted number of specific combinations were detected within the ocular surface compartments. The most prevalent combinations were based on the simultaneous presence of CD73, CD90, CD105, and CD166, and alternating expression of CD146 and CD34, which in total represented 88.4% of all cells (Figure 3). Taking into account also the variants negative for the CD166, the proportion of major phenotypes reached on average 94.5%. The representation of the major variants differed between particular ocular surfaces, with cornea, limbus, and sclera averaging, 96.4%, 90.0%, and 97.1%, respectively. Limbus clearly harbored, when compared to the other ocular compartments, the highest proportion of minor variants outside the hallmark triple combination of CD73+CD90+CD105+, and thus in terms of variant distribution appeared as the most heterogeneous segment. This conclusion could also be corroborated when looking at another, more qualitative, parameter, which was the frequency of the different minor variant types.

Regarding particular major variants, irrespective of the ocular location, the CD73+CD90+CD105+CD166+CD146–CD34– appeared to be the most prevalent and the CD73+CD90+CD105+CD166–CD146–CD34– the least prevalent phenotypes. With respect to the individual ocular compartments, it is of interest that limbus exhibited highest proportion of the pericyte-like CD146+CD34– phenotype. This phenotype was found mostly on the background of the CD73+CD90+CD105+CD166+ combination, but also with much less frequency in the context of the hallmark triple positivity for CD73, CD90, and CD105.

### Co-expression of CD146 and CD34

Since the CD146 and CD34 have previously been shown to delineate important stem cell variants related to pericytic, advential, and intermediate populations,^[25]^ the hOSSCs were subsequently analyzed for their co-expression. No significant differences were observed among the three ocular compartment in the proportion of double-positive or -negative, or CD34 single-positive cells (Figure 4A). This could not be confirmed with the reference cells, probably as a consequence of remarkably high CD146 expression by EMSCs. Interestingly, however, the hOSSCs were less uniform regarding the expression of the pericyte-like stromal cell phenotype featuring the CD146+CD34– profile. Here, the limbal compartment surpassed the adjacent segments by more than two-fold, and similar pattern could be seen with the EMSCs within the reference cell group. The superior prevalence of pericytic phenotype in the limbus compartment is also clearly illustrated from the individual donor perspective (Figure 4B).

##  DISCUSSION

Our results demonstrated that the most prevalent pattern in the hOSSC cultures featured a canonical quadruple CD73, CD90, CD105, and CD166 co-expression, which is reminiscent of a general MSC profile.^[26]^ The frequent occurrence of variants with this signature may have a biological significance, since some of these markers were found to be involved in the corneal wound healing.^[27]^ Regarding the two minor antigens, the CD146 and CD34, they were not co-expressed consistently together with the canonical repertoire, and their levels were influenced by a substantial donor-related variability. There was, however, an obvious trend toward low CD34 expression in the limbal cells, which was also well in accordance with the phenotype of the reference cells. Although it is generally believed that CD34 expression is related to hematopoietic origin, more recent evidence demonstrates CD34 is also expressed by nonhematopoietic progenitor cells,^[28]^ including neural crest-derived precursors from cornea.^[29]^ Therefore, the origin and function of CD34+ cells from different ocular surface stromal compartments is of interest and requires further exploration. As for the CD146, it has been reported to be expressed at a high level in EMSCs^[30]^ and from the functional point of view, it has been proposed to be associated with high trilineage potency.^[31]^ But in the context of the limbal niche, it is plausible that this phenotypical hallmark plays an important role in corneal homeostasis. This stems from previous findings that the irradiated fibroblasts isolated from the limbus supported better the growth of epithelial progenitors than the fibroblasts isolated from the central cornea or sclera^[32]^ and that these cells typically featured the CD146.^[33]^

Another interesting result was provided by a comparative analysis of the three ocular surface compartments for the presence of distinctive MSC subtypes, including the perivascular MSC subpopulations, namely pericyte– (CD146+ CD34–)^[31]^ and adventitial-like cells (CD146–CD34+),^[34]^ as well as a group of cells at their intermediate stage (CD146+CD34+), which have been suggested to possess a proliferative capacity.^[25]^ Strikingly, the pericytes were highly represented in the limbal compartment, irrespective of an inter-donor variability, which is known to impart a substantial confounding effect.^[13]^ Since the pericyte phenotype is CD34 negative, the implication is that this marker is in the limbal compartment encountered at invariably low levels, as noted above. The presence of pericyte-like cells in limbal stroma in this study is in line with previous reports,^[35,36]^ nevertheless, it is for the first time that we are showing that the limbal stromal cultures harbor a significantly higher proportion of pericyte-like cells than their donor-matched corneal or scleral counterparts. This finding thus indicates that an elaborate limbal MSC niche entailing substantial cellular complexity and intricate cross-talk is essential for a normal self-renewal of the LESCs. Such networking is reminiscent of the role the MSC niche plays in supporting the homeostasis of hematopoiesis.^[37]^

It is also worthwhile to mention that this is the first time that the presence of perivascular MSCs has been documented in avascular central corneal stroma. Nonetheless, it is necessary to state that the association of these cell types with angiogenesis through examination of specific functional markers, such as NG2, SMΑ, desmin, vimentin, and PDGFR-β, was not done in this study. Thus, in light of our data, it appears that the classical paradigm associating specific phenotypes with perivascular microenvironment needs to be reconsidered, and it will be interesting to see in the future what biological role these cells play in the cornea.

In conclusion, the hOSSCs represent a greatly heterogeneous population that seems to play an important role in the maintenance of ocular surface and most significantly, the cornea. Better understanding of these cells undoubtedly holds promise for improvement of the *in vitro* protocols, which will ultimately spur the development of new generation of ocular surface stem cell-based products for sight-threatening corneal diseases such as limbal stem cell deficiency or corneal scarring.

##  Conflicts of Interest

There are no conflicts of interest.
